# Human papillomavirus 16E6/E7 activates autophagy via Atg9B and LAMP1 in cervical cancer cells

**DOI:** 10.1002/cam4.2351

**Published:** 2019-06-18

**Authors:** Chen Tingting, Yang Shizhou, Zhang Songfa, Xu Junfen, Lu Weiguo, Cheng Xiaodong, Xie Xing

**Affiliations:** ^1^ Department of Gynecologic Oncology Women's Hospital, Zhejiang University School of Medicine Hangzhou China

**Keywords:** Atg9B, autophagy, cervical cancer, HPV, LAMP1

## Abstract

**Backgrounds:**

Although the role of high‐risk human papillomavirus (HPV) E6 and E7 in cellular malignant transformation has been elucidated， the function of both genes in cellular homeostasis is still unknown. Autophagy functions in maintenance of cellular homeostasis play a key role in the initiation and development of cancer and infectious disease.

**Methods:**

Cervical cancer cell lines SiHa and CaSki were utilized in this study.

**Results:**

We found that HPV 16E6/E7 (16E6/E7) downregulation inhibited autophagy, and consequently suppressed cell proliferation and promoted early apoptosis. Transcriptome sequencing demonstrated that Atg9B and LAMP1 were downregulated in 16E6/E7 knockdown cells. Gene function experiments revealed that 16E6/E7 downregulation depressed Atg9B and LAMP1, and Atg9B and LAMP1 overexpression compensated, at least partially, autophagy blockage induced by 16E6/E7 knockdown. Immunoprecipitation assay showed that 16E7 interacted with Atg9B and dual‐luciferase reporter system revealed that 16E6 most likely regulated −1750 to −2000 nt in Atg9B and −1800 to −2000 nt in LAMP1 promoter region.

**Conclusions:**

Our findings verified that 16E6/E7 activated autophagy via accelerating autophagosome formation and degradation, and Atg9B and LAMP1 were involved in the process of 16E6/E7 modulating autophagy, suggesting that targeting autophagy may be a potential approach in cervical cancer therapeutics.

## 1 INTRODUCTION

Cervical cancer has significantly declined in developed countries due to popularized cancer screening, but it still ranks the third common women's cancer in the world and is the leading cause of cancer death in less developed countries.^1^, ^2^, ^3^ The limited efficacy of the current therapeutic methods, including surgery, chemotherapy, and radiotherapy, may contribute to the high death rate in the patients with advanced disease.^3^ To find out the pivotal events and regulatory molecules in cancer progression may be a key approach to achieve precision treatment toward cervical cancer, thereby improving the prognosis of patients with advanced stage.

Autophagy is regarded as a fundamental mechanism to maintain cellular homeostasis, but acts as a “double‐edged sword.” Proper‐activated autophagy provides energy to support cellular metabolism when cells live under stress, while over‐activated autophagy leads to cell death.^4^, ^5^, ^6^, ^7^, ^8^, ^9^ It has been reported that some kinds of viruses, such as hepatitis B virus (HBV), human T‐lymphotropic virus‐1 (HTLV‐1), Epstein‐Barr virus (EBV), and others, can utilize certain autophagy‐related proteins to facilitate a long‐term survival in host cells.^10^, ^11^, ^12^, ^13^ In a sense, the status of host autophagy activation determines the fate of virus. Regulation of autophagic activity has become a promising therapeutic strategy though its exact mechanism involving in the process of some diseases remains unclear today.^14^, ^15^

Accumulated evidences have revealed that cervical cancer is one kind of virus‐associated cancers. Persistent infection of high‐risk human papillomavirus (HR‐HPV) is an essential event during cervical cancer initiation and progression, of those, HPV16 is the most common genotype, accounting for over 60% cervical cancers.^16^, ^17^, ^18^, ^19^, ^20^, ^21^ Classical theory considers that HR‐HPV possesses the ability to integrate viral genome into the host DNA, which produces the overexpression of early viral oncoprotein E6 and E7 and results in the initiation and progression of cervical cancer via E6‐p53 and E7‐pRB signaling pathway, respectively.^22^, ^23^, ^24^, ^25^, ^26^ Although the role of HPV in inducing malignant transformation and promoting malignant biobehavior of infected cells has been well recognized, the effect of virus on maintaining cellular homeostasis, via modulating autophagy, is still uncovered, except for a few studies in which HPV was shown to modulate autophagy in cervical cells. For example, HPV16 pseudovirus activates PI3K/Akt/mTOR pathway and inhibits autophagy in the early stages of virus‐host interaction in order to increase the infectious efficacy.^27^ The overexpression of HPV16 E7 oncoprotein induces an autophagy‐like process in human foreskin keratinocytes and cells are prone to death upon serum deprivation.^28^ The depletion of HPV16 E7 in W12 cells, a kind of immortalized precancer cell line, induces cellular senescence, although autophagy is activated.^29^ Obviously, the results from those limited reports are contradicted.

Here, we knocked down HPV16‐E6/E7 (16E6/E7) expression in cervical cancer SiHa and CaSki cells and enforcedly overexpressed 16E6/E7 in HEK293 cells, and observed the alteration of autophagy marker protein LC3‐II expression and autophagy flux. We then screened and confirmed target genes of 16E6/E7 by transcriptome sequencing, bioinformatics, and gene function experiments. The aim of the study was to verify the ability of HPV16 oncoproteins to maintain proper‐activated autophagy via their target genes, thereby to provide the evidence for targeting autophagy as an approach in cervical cancer therapeutics.

## 2 MATERIAL AND METHODS

### 2.1 Cell culture and reagents

Human cervical cancer cell lines SiHa, CaSki, and HEK293 were obtained from the American Type Culture Collection (ATCC) and maintained in DMEM (Coring, 10‐013) or RPMI 1640 (Corning, 10‐040‐CVR) medium, supplemented with 10% fetal bovine serum (FBS) at 37°C and 5% CO_2_.

Bafilomycin A1 (Sigma‐Aldrich, B1793), rapamycin (Sigma‐Aldrich, R0395), chloroquine (Sigma‐Aldrich, C6628), 3‐MA (Sigma‐Aldrich, M9281), and EBSS (Sigma‐Aldrich, E2888) were prepared as instructed.

### 2.2 Western blotting

The appropriate amounts of proteins were applied and subjected to SDS‐PAGE. The membranes were incubated with primary antibodies at 4°C overnight after proteins were transferred to 0.22 µm PVDF membranes (ISEQ00010, Millipore) and blocked with 5% nonfat dry milk in TBST. The bands were detected with an EZ‐ECL kit (BI biological industries, 20‐500‐120) in Imagequant LAS400 mini (GE Healthcare) equipment after incubation with secondary antibodies.

Primary antibodies to Beclin1 (cell signaling technology, 3738), LC3 (Sigma‐Aldrich, L7543), p53 (Abcam, ab1101), pRB (Abcam, ab32015), Atg5 (Cell Signaling Technology, 8540), Atg16L1 (Sigma‐Aldrich, A7356), LAMP1 (Abcam, ab24170), SPATA18 (Abcam, ab107702), Atg9B (Thermofisher Scientific, PA5‐20998), RGS19 (Abcam, ab72085), HA (Sigma‐Aldrich, H9656), Flag (Sigma‐Aldrich, F7425), GAPDH (Abcam, ab9485), ACTB (Abcam, ab8224), Histone H3 (Abcam, ab1791), and Na‐K ATPase (Abcam, ab76020) were applied in our study. HPR‐labeled secondary anti‐mouse (115‐035‐003) and anti‐rabbit (111‐035‐003) antibodies were purchased from Jackson Immunoresearch Laboratories.

Subcellular extraction was collected with subcellular protein fraction kit for cultured cell (Thermofisher Scientific, 78840).

### 2.3 Transfection

siRNA and scrambled siRNA‐negative control were synthesized by GenePharma (Shanghai, China), as shown in Table S1. Plasmids were constructed by GeneScript (Nanjing, China). Atg9B Double Nickase Plasmid was purchased from Santa Cruz Biotechnology (sc‐406894‐NIC). For transient transfection, cell lines were seeded at 60% confluency, and transfection experiments were performed with siRNA at a final concentration of 100 nmol/L using DharmaFECT 1 transfection reagent (Dharmacon) or 1ug plasmid using Roche HP DNA Transfection Reagent (Roche) according to the manufacturer's guidelines. After overnight incubation, the culture medium was replaced with fresh Dulbecco's modified Eagle's medium containing 10% FBS before further study. The expression level in the transfected cells at 48 hours posttransfection was directly confirmed by qRT‐PCR.

### 2.4 RNA extraction and qRT‐PCR

Total RNA containing mRNA was extracted using TRIzol reagent (Invitrogen, Carlsbad, CA, USA) following the manufacturer's instructions. cDNA was synthesized with the PrimeScript RT reagent Kit (TaKaRa Otsu, Shiga, Japan). qRT‐PCR analyses for mRNA of interest were performed using SYBR Premix Taq (TaKaRa) as previously reported. GAPDH was identified as a suitable internal control for human cervical tissue samples. The primers used are shown in Table S2.

### 2.5 Direct immunofluorescence

SiHa cells were transfected with Ad‐LC3 and Si‐16E6/E7 or negative control for 48 hours and transferred to coverslips. The cells were treated with EBSS 100% for 2 hours and/or 50 µm CQ for 3h after adhering to coverslips. Nuclei were stained with 4′,6‐diamidino‐2‐phenylindole (DAPI, Sigma‐Aldrich, D9542) followed by fixing with 4% paraformaldehyde (Sigma‐Aldrich, 158127) and permeabilized with 0.5% Triton X‐100 (Solarbio, T8200‐100) in PBS (Corning, R21‐040‐CV). Images were taken with a spinning disk confocal fluorescence microscope (Olympus). The amounts of LC3‐II‐positive puncta in 20 cells for each group were counted using the ImageJ and the numbers of green and red dots per cell were calculated.

### 2.6 Transcriptome sequencing and bioinformatics analysis

Transcriptome sequencing and KEGG and GO analyses were performed by RiboBio Co., Ltd.

The sequences of 2 kb nucleotides prior to the first exon of Atg9B and LAMP1 genes were searched in the NCBI database. The sequences were submitted to the JASPAR database to find out transcriptional factors and transcriptional elements, respectively. All these transcriptional elements were blast with 16E6 to predict the binding sites for 16E6 regulating Atg9B and LAMP1 genes.

### 2.7 Luciferase reporter assay

For luciferase reporter experiments, the sequences of 2 kb prior to the first exon of Atg9B and LAMP1 genes were searched in the NCBI database and constructed into the pGL3‐Basic vector (Promega, Madison, WI, USA) by GeneScript (Nanjing, China). Cotransfection of 16E6 plasmid and the reporter gene was performed using Roche HP DNA Transfection Reagent (Roche) following the manufacturer's manual. Briefly, assayed cells were grown to about 60% confluence in a 96‐well tissue culture plate. Cells were cotransfected with pGL3‐Basic promoter and tested plasmids. After 48 hours, the luciferase activities were assessed using the Dual‐Glo Luciferase Assay System (Promega) by measuring the intensity of chemiluminescence in a luminometer (Thermo Fisher Scientific, Waltham, MA, USA). The experiments were performed in triplicate and repeated at least three times with negative controls.

### 2.8 Cell proliferation analysis

To evaluate cell proliferation, SiHa (1 × 10^4^ cells per well) and CaSki cells (6 × 10^3^ cells per well) were separately plated in 96‐well plates and transfected on the following day with siRNA or treated with autophagy regulatory drugs. At 0, 24, 48, 72, and 96 hours after transfection, cell viability was identified using the Cell Counting Kit‐8 (CCK‐8, Dojindo laboratories, CK04). The Varioskan Flash microplate reader (Thermo Scientific) was used for spectral analyses. And, the proliferation curve was generated based on absorbance and time.

### 2.9 Apoptosis assay

For apoptosis assays, 48 hours posttransfection or exposure to autophagy regulatory drugs, adherent cells were harvested and washed twice with phosphate‐buffered saline. Annexin‐V in combination with propidium iodide (Biouniquer) was added to the cells and samples were analyzed within 30 min after staining. Quantification of fluorescence was analyzed by flow cytometry (Beckman Coulter, Fullerton, CA, USA).

### 2.10 Statistical analysis

Statistics were assessed using software packages SPSS version 19.0 (WPSS Ltd, Surrey, UK). The results were presented as mean ± SE/SD Relative quantification of mRNA expression was calculated with the 2^−ΔΔ^Ct method. Student's *t* test was used to analyze the differences between two groups. All *P*‐values were two‐sided and *P* < 0.05 were regarded as significant.

## 3 RESULTS

### 3.1 16E6/E7 maintains cellular autophagy

**Figure 1:**
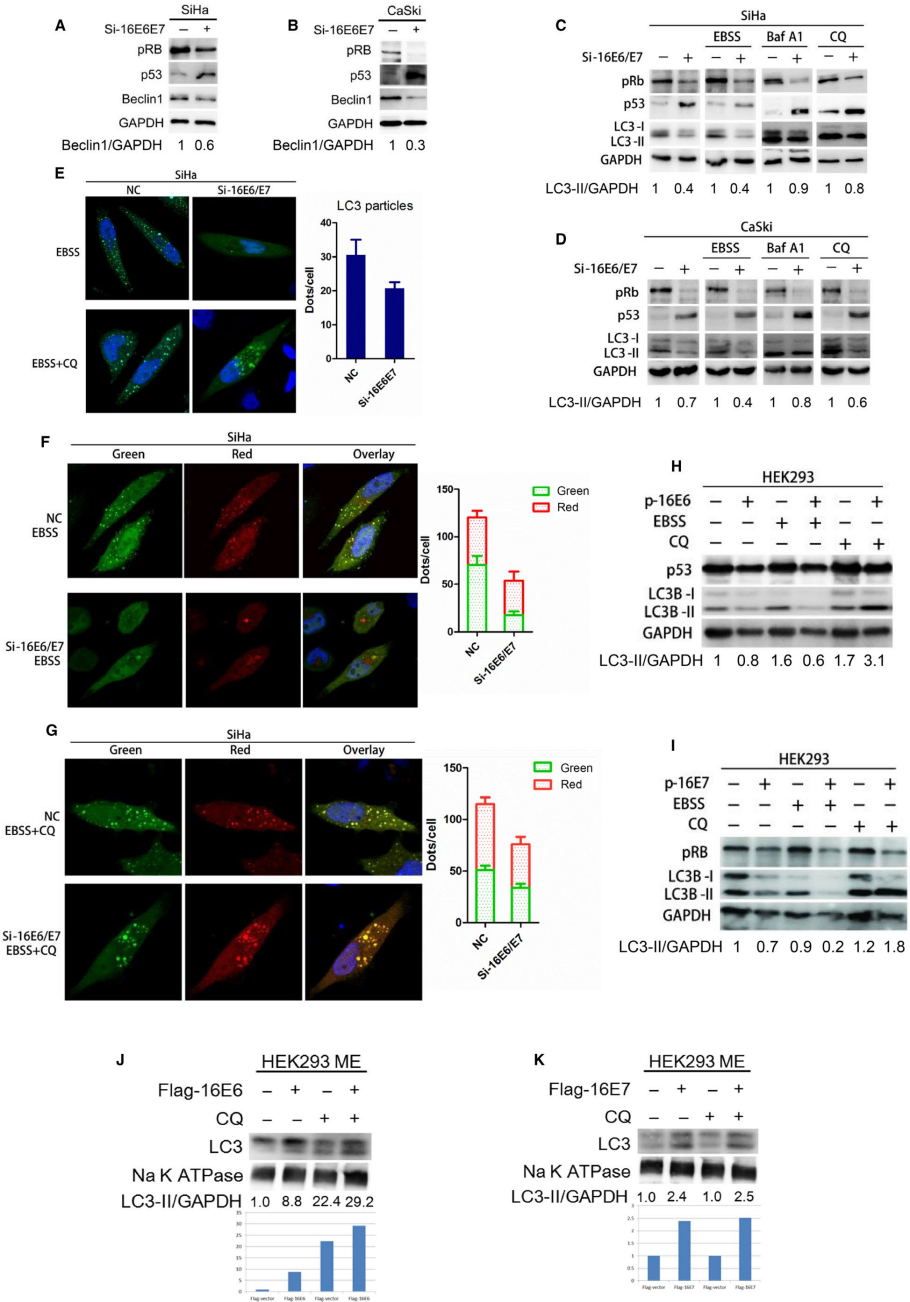
16E6/E7 maintains autophagy in cervical cancer cells. A, Beclin1 expression was decreased after 16E6/E7 knockdown in SiHa cells. B, Beclin1 expression was decreased after 16E6/E7 knockdown in CaSki cells. C, LC3‐II expression was decreased after 16E6/E7 knockdown in SiHa cells, with or without EBSS, Baf A1, or CQ. D, LC3‐II expression was decreased after 16E6/E7 knockdown in CaSki cells, with or without EBSS, Baf A1, or CQ. E, LC3‐II green dots were decreased when SiHa cells were transfected with Si‐16E6/E7, the number of dots in SiHa with EBSS and CQ was shown as histogram, *P* < 0.05. F, Green and red dots of LC3‐II were decreased when SiHa cells were cotransfected with Ad‐LC3 and Si‐16E6/E7, with EBSS exposure, *P* < 0.05. G, Green and red dots of LC3‐II were decreased when SiHa cells were cotransfected with Ad‐LC3 and Si‐16E6/E7, with EBSS and CQ exposure, *P* < 0.05. H, LC3‐II expression decreased when overexpressing 16E6 in HEK293 cells, with or without EBSS, but accumulated when overexpressing 16E6 in HEK293 cells with CQ. I, LC3‐II expression decreased when overexpressing 16E7 in HEK293 cells, with or without EBSS, but accumulated when overexpressing 16E7 in HEK293 cells with CQ. J, LC3‐II in membrane was elevated in overexpressing 16E6 cells with CQ exposure. K, LC3‐II in membrane was elevated in overexpressing 16E7 cells, with or without CQ exposure

RNA interference (RNAi) was applied to knockdown 16E6/E7 expression in HPV16‐positive cervical cancer cell line. RT‐RCR assay was carried out to measure the efficiency of Si‐16E6/E7 in SiHa and CaSki, respectively. 16E6/E7 transcript abundance was significantly decreased when cells were transfected with Si‐16E6/E7 (Figure S1A and B). A pulse‐chase experiment with chloroquine over a 1 hour period was conducted, and LC3‐II expression and lipidation were decreased in 16E6/E7 low‐expressed SiHa cells cultured with CQ for 3 hours (Figure S1C). The expressions of p53 and pRB, downstream molecules of 16E6 and 16E7, respectively, were tested to confirm the interference efficiency again. Si‐16E6/E7 significantly eliminated the effect of 16E6‐promoting p53 degradation and 16E7‐inducing pRB accumulation in cervical cancer cells. Autophagy‐related protein Beclin1 was decreased after 16E6/E7 knockdown in SiHa and CaSki cells (Figure 1A and 1). Further, the expression of LC3‐II, an autophagy marker protein, was decreased in 16E6/E7 knockdown cells with or without exposure to starvation (100% EBSS). Next, Bafilomycin A1 (Baf A1) and Chloroquine (CQ), two autophagic blockers, were used to confirm which stage of autophagy, autophagosome formation, or degradation was affected. As shown in Figure 1C and 1, LC3‐II expression was less accumulated in 16E6/E7 knockdown cervical cancer cells than that in control cells with Baf A1 or CQ exposure. Similarly, laser scanning confocal microscopy showed that the numbers of LC3 green dots were reduced in 16E6/E7 knockdown cells with EBSS and CQ exposure (Figure 1E). mRFP‐GFP‐LC3 double‐labeled adenovirus (Ad‐LC3) was introduced to monitor autophagy flux. With Ad‐LC3, both green and red dots of LC3 were decreased in 16E6/E7 knockdown cells following starvation induction, with and without CQ (Figure 1F, 1, and Figure S1D). Our results suggested that 16E6/E7 downregulation contributes to autophagy inhibition, with and without autophagic blocker.

Contrarily, HEK293 cells transfected with 16E6 or 16E7 plasmid were used as a model that overexpressed 16E6 or 16E7, and transcript abundance was tested in RT‐PCR, respectively (Figure S1E). The expression of LC3‐II was decreased in both 16E6‐ and 16E7‐overexpressed HEK293 cells with and without starvation induction, but increased when autophagic blocker CQ was added (Figure 1H and 1). As LC3‐II is mainly a kind of membrane‐associated protein, membrane extraction (ME) was collected from HEK293 cells transfected with 16E6 or 16E7 plasmid for 48h. LC3‐II in membrane was elevated in 16E6 and 16E7 overexpressed cells, with and without CQ exposure (Figure 1J and 1). Our results suggest that both 16E6 and 16E7 accelerate autophagosome formation and degradation, consequently promoting autophagic flux in cells. Combined with the results observed in cervical cancer cells with 16E6/E7 knockdown, we speculate that 16E6/E7 plays a positive role on autophagy activity in cervical cancer cells, via accelerating autophagy flux in the initiation or elongation stage of autophagy.

### 3.2 Blocked autophagy inhibits the viability of cervical cancer cells

**Figure 2:**
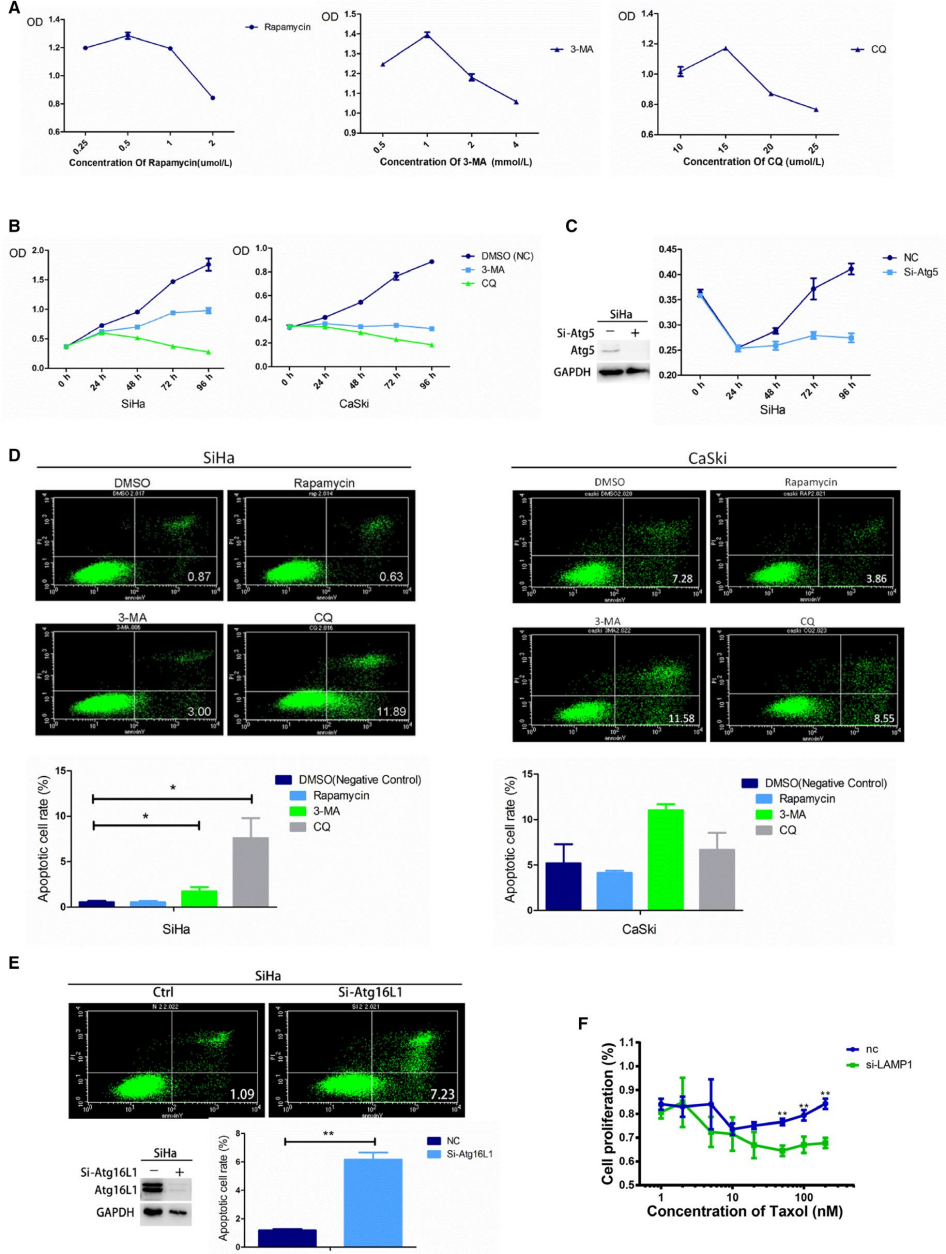
Blocked autophagy inhibits the viability of cervical cancer cells. A, 0.5 µmol/L rapamycin, 1 mmol/L 3‐MA, and 15 µmol/L CQ were selected as optimal experimental concentrations. B, Cell proliferation was inhibited when SiHa and CaSki cells were cultured with 3‐MA or CQ. C, Cell proliferation was inhibited when autophagy was impaired by Atg5 knockdown in SiHa cells. D, Cell early apoptosis was slightly inhibited when cultured with rapamycin, but was significantly elevated with 3‐MA and CQ in SiHa and CaSki cells. E, Cells early apoptosis was induced when autophagy impaired by Atg16L1 knockdown in SiHa cells

Autophagic inducer Rapamycin, autophagic inhibitor 3‐MA, and autophagic blocker CQ were used, respectively, to examine the effect of autophagy on cell viability, and 0.5 µmol/L rapamycin, 1 mmol/L 3‐MA, and 15 µmol/L CQ were selected as optimal experimental concentrations by CCK‐8 assay (Figure 2A). Cell proliferation was inhibited after cells were cultured with 3‐MA or CQ in both SiHa and CaSki cell lines (Figure 2B). The result was similar when autophagy was blocked by Atg5 knockdown (Figure 2C). In addition, cell early apoptosis was slightly inhibited after cervical cancer cells cultured with rapamycin, but was significantly elevated with 3‐MA and CQ, detected by flow cytometry (Figure 2D). The results were similar after autophagy inhibited by Atg16L1 knockdown (Figure 2E). Our results suggest that impaired autophagy inhibits the viability in cervical cancer cells.

### 3.3 Atg9B and LAMP1 were candidate genes involving in 16E6/E7 modulating autophagy

**Figure 3:**
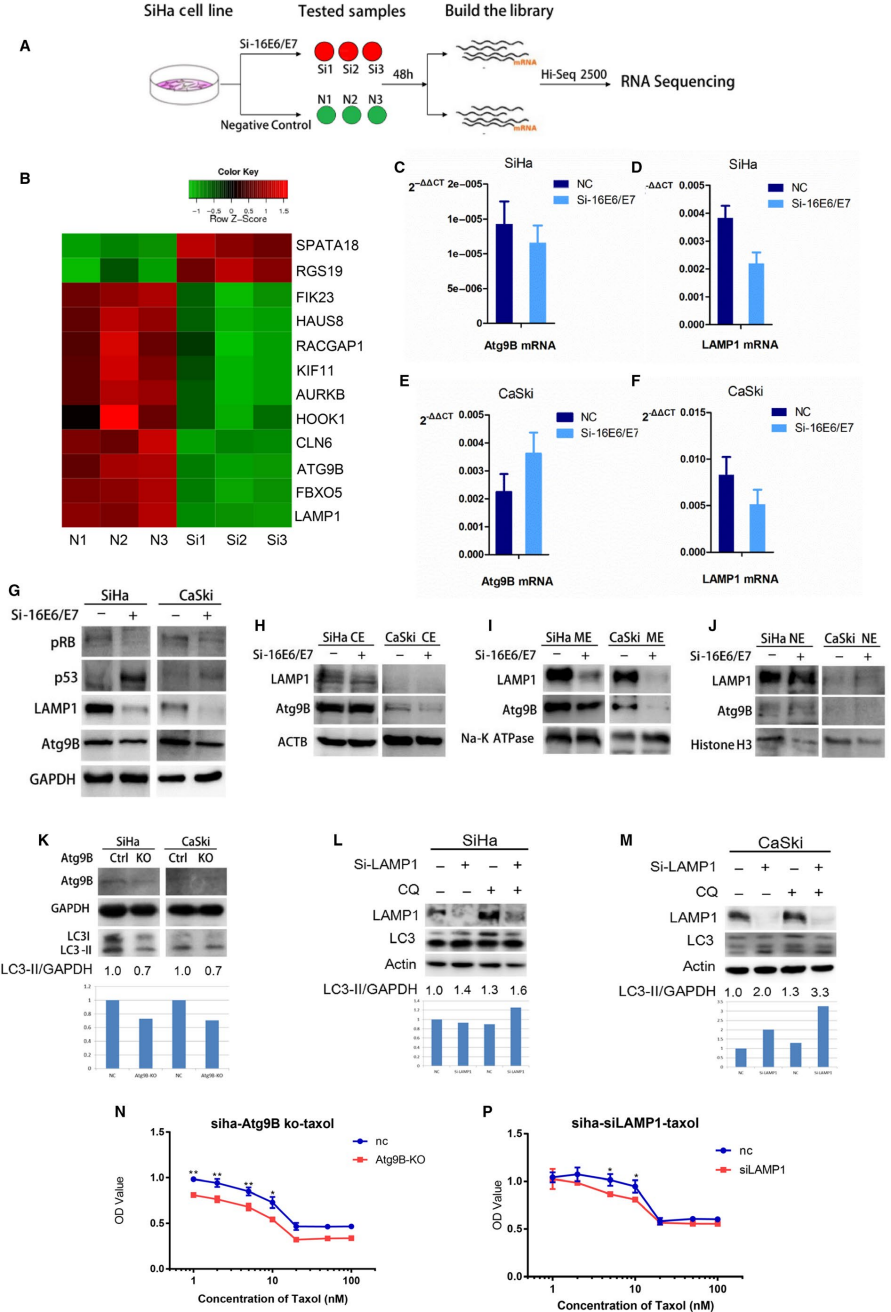
Atg9B and LAMP1 were candidate genes involving in 16E6/E7 modulating autophagy. A, The process of RNA‐sequencing. SiHa cells transfected with Si‐16E6/E7 or negative RNAi (3 samples for each group) were prepared to build cDNA library. RNA‐sequencing was performed in a Hiseq 2500 platform. B, Autophagy‐ and apoptosis‐associated genes were selected to be candidate downstream regulators of 16E6/E7 and showed in a heatmap. C, The expression of Atg9B mRNA was downregulated in SiHa cells transfected with Si‐16E6/E7. D, The expression of LAMP1 mRNA was downregulated in SiHa cells transfected with Si‐16E7/E7. E, The expression of Atg9B mRNA was downregulated in CaSki cells transfected with Si‐16E6/E7. F, The expression of LAMP1 mRNA was upregulated in CaSki cells transfected with Si‐16E6/E7. G, The expression of LAMP1 and Atg9B protein was downregulated in SiHa and CaSki cells transfected with Si‐16E6/E7. H, I, J, Atg9B, and LAMP1 were mainly detected in ME and decreased after 16E6/E7 knockdown in both SiHa and CaSki cells. K, LC3‐II was decreased when Atg9B knockout by double nickase plasmid in SiHa and CaSki cells. L, LC3‐II was accumulated when LAMP1 knockdown in SiHa cells with CQ exposure. M, LC3‐II was accumulated when LAMP1 knockdown in CaSki cells, with or without CQ exposure. N, (P) Atg9B knockout or LAMP1 knockdown elevated the chemosensitivity of SiHa cells

SiHa cells transfected with Si‐16E6/E7 and negative RNAi (three samples for each group) were prepared to build cDNA library, respectively. Transcriptome sequencing was performed by RiboBio Co., Ltd in a Hi‐seq 2500 platform (Figure 3A). Totally, 586 genes were differentially expressed, including 193 overexpressed and 393 down‐expressed, by DeSeq analysis (Figures S2 and S3). All 586 genes were categorized according to KEGG (Kyoto Encyclopedia of Genes and Genomes) pathways and GO (Gene Ontology) analysis. Three top enriched pathways were metabolism, cell cycle, and cancer signaling pathway (Figure S4) and six top enriched GO cellular components were organelle, intracellular organelle, organelle part, nucleus, intracellular organelle part, and intracellular non‐membrane‐bounded organelle (Figure S5). Those genes associated with autophagy and apoptosis were considered to be candidate downstream regulators of 16E6/E7 (Figure 3B). Among them, SPATA18 (spermatogenesis associated 18), RGS19 (regulator of G‐protein signaling，RGS19), Atg9B (Autophagy‐related 9B), and LAMP1 (lysosomal associated membrane protein 1), which had been reported to participate directly or indirectly in autophagy, were selected to be verified by RT‐PCR and western blot. RT‐PCR confirmed that SPATA18 and RGS19 mRNA were upregulated and Atg9B and LAMP1 mRNA were downregulated in SiHa cells with Si‐16E6/E7 for 48 hours (Figure 3C, 3D, Figure S6A and B). But, LAMP1 mRNA was downregulated and other three mRNAs were upregulated in Caski cells transfected with Si‐16E6/E7 (Figure 3E, 3F, Figure S6C and D). All four proteins mentioned above were decreased in both SiHa and CaSki cells (Figure 3G and Figure S6E). So, only LAMP1 revealed an accordant change of expression of mRNA and protein in both cell lines. Compared to the uncertainty of RGS19 and SPATA18 in modulating autophagy, Atg9B is a kind of autophagy‐related protein which can transport autophagosome and LAMP1 is associated with autophagosome degradation. Further, cytoplasmic extraction (CE), membrane extraction (ME), and nuclear extraction (NE) were separately collected after SiHa and CaSki cells were transfected with Si‐16E6/E7 for 72 hours. Atg9B and LAMP1 were mainly detected in ME and decreased after 16E6/E7 knockdown in both SiHa and CaSki cells (Figure 3H‐J). Thus, Atg9B and LAMP1 were selected as two candidates for further gene function study.

To validate the effect of Atg9B and LAMP1 on autophagy, they were enforcedly downregulated in SiHa and CaSki cells, respectively. As we expected, LC3‐II was slightly decreased after Atg9B knockout by double nickase plasmid (Figure 3K), due to failure to knockdown by RNAi, but accumulated after LAMP1 knockdown by RNAi, especially when cells were cultured with autophagic blocker CQ (Figure 3L and 3). Since cells with autophagy may present chemoresistance, we examined the effect of Atg9B or LAMP1 inhibition on chemoresistance to taxol exposure in SiHa and CaSki cells, and found that Atg9B knockout or LAMP1 knockdown elevated the chemosensitivity of SiHa cells (Figure 3N and 3). Our results suggest that Atg9B and LAMP1, as candidate downstream regulators of 16E6 and 16E7, play a positive role in promoting autophagy flux, as 16E6 and 16E7 do, in cervical cancer cells.

### 3.4 Atg9B and LAMP1 are involved in 16E7 modulating autophagy in cervical cancer cells

**Figure 4:**
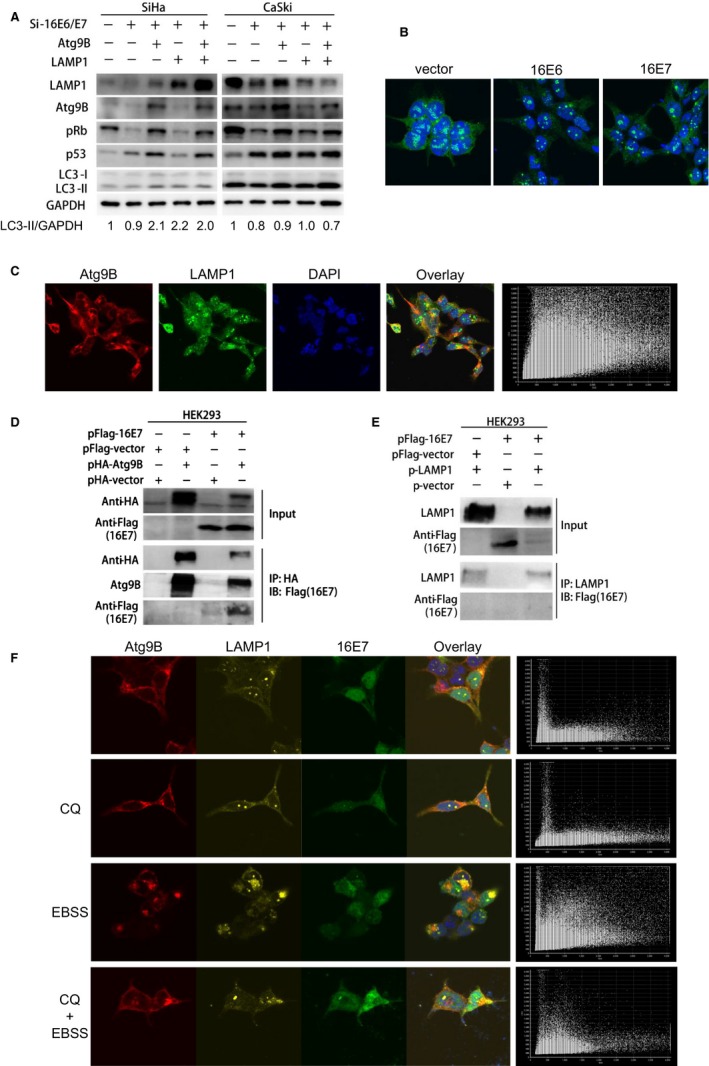
Atg9B and LAMP1 are involved in 16E7 modulating autophagy in cervical cancer cells. A, LC3‐II was accumulated when Atg9B were overexpressed and reduced when LAMP1 was overexpressed in 16E6/E7 knockdown cells, compared to the control group. B, LAMP1 was gathered as several clustered dots, while larger but less LAMP1 dots were formatted in HEK293 cells with overexpressed 16E6 or 16E7. C, Atg9B with red and LAMP1with green fluorescence were colocalized (Pearson's coefficient 0.726). D, 16E7 interacted with Atg9B. E, 16E7 do not interact with LAMP1. F, Atg9B was colocalized with LAMP1 in HEK293 cell with overexpressed 16E7 (Pearson's coefficient 0.457, 0.525, 0.717, and 0.456, respectively)

To confirm the role of Atg9B and LAMP1 in 16E6E7 modulating autophagy, rescue experiment was carried out. LC3‐II was accumulated after Atg9B was overexpressed and reduced after LAMP1 was overexpressed in 16E6/E7 knockdown cells, compared to the control group (Figure 4A). Our results suggest that Atg9B and LAMP1 overexpressions compensate, at least partially, the autophagic blockage induced by 16E6E7 knockdown in cervical cancer cells.

Immunofluorescence assay was used to show the positional relationship of Atg9B and LAMP1 in cells. LAMP1 was gathered as several clustered dots, while larger but less LAMP1 dots were formatted in HEK293 cells with overexpressed 16E6 or 16E7 (Figure 4B). Then, the positional relationship between Atg9B and LAMP1 was observed. Red fluorescence of Atg9B and green fluorescence of LAMP1 were colocalized in HEK293 cells with overexpressed Atg9B and LAMP1 (Figure 4C), but there was no interaction between Atg9B and LAMP1 by immunoprecipitation analysis. After Flag‐16E7 and HA‐Atg9B plasmids were transfected into HEK293 cells, immunoprecipitation analysis showed the interaction between 16E7 and Atg9B, but not LAMP1 (Figure 4D and 4). Further, 16E7, Atg9B, and LAMP1 plasmids were simultaneously transfected into HEK293 cells, laser confocal scanning microscopy showed that Atg9B was colocalized with LAMP1 in cells regardless of starvation induction or CQ exposure (Figure 4F). However, no interaction between GFP‐16E6 and Atg9B or LAMP1 was observed by immunoprecipitation analysis. Thus, our results together indicate that 16E7, but not 16E6, interacts with Atg9B and Atg9B colocalizes with LAMP1, consequently activating autophagy, suggesting that Atg9B and LAMP1 are involved in 16E7 modulating autophagy.

### 3.5 16E6 positively regulates the transcription of Atg9B or LAMP1 genes

**Figure 5:**
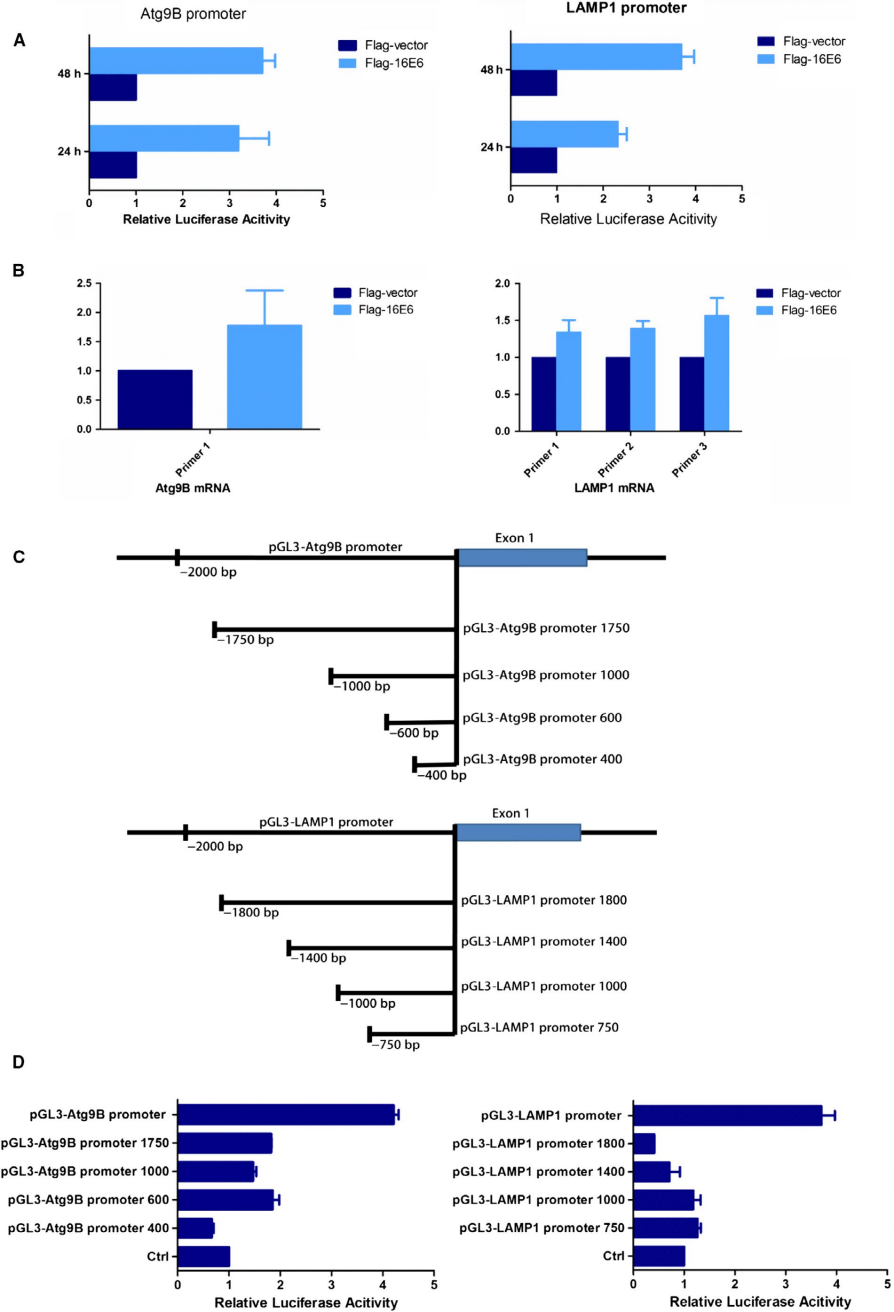
16E6 positively regulates the transcription of Atg9 and LAMP1 genes. A, 16E6 positively regulated the transcription of Atg9 and LAMP1. B, The expressions of Atg9 and LAMP1 mRNA were increased when 16E6 was overexpressed. C, Different luciferase plasmids of Atg9 or LAMP1 promoter regions were truncated according to the predicted results. D, The most responsible sequences for 16E6 may be most likely located in‐1750 to −2000 nt in Atg9B promoter and −1800 to −2000 nt in LAMP1 promoter region

Because 16E6 did not interact with Atg9B or LAMP1, it was necessary to uncover the mechanism by which 16E6 modulating Atg9B or LAMP1. 16E6 acted as transcriptional regulators as previously reported^30^, ^31^ and was mainly located in nucleus as observed in this study by immunofluorescence. Thus, it is probable for 16E6 to modulate Atg9B and LAMP1 at the transcriptional level. According to the NCBI database, sequences of 2 kb nucleotides prior to the first exon of the two genes were constructed, respectively, into pGL3‐Basic vector as promoter region that 16E6 probably bind to. Dual‐luciferase reporter assay showed that 16E6 positively regulated the transcription of Atg9B and LAMP1 in HEK293 cells (Figure 5A). The results were verified by RT‐PCR that both Atg9B and LAMP1 mRNA were increased when 16E6 was overexpressed (Figure 5B). Further，the binding sites for 16E6 in the promoter region of Atg9B or LAMP1 gene were predicted in the JASPAR database, respectively, and −720 to −710 nt, −1085 to −1075 nt, and −1857 to −1860 nt in Atg9B promoter and −700 to −690 nt, −1265 to −1255 nt, and −1880 to −1870 nt in LAMP1 promoter were found to be most likely sites for 16E6 to bind to (Tables S3 and S4). Different luciferase plasmids of Atg9B or LAMP1 promoter regions were truncated according to the predicted results (Figure 5C). The most likely responsible sequences for 16E6 may be located in −1750 to −2000 nt in Atg9B promoter and −1800 to −2000 nt in LAMP1 promoter (Figure 5D). Thus, our results suggest that 16E6 regulates Atg9 or LAMP1 expression positively at the transcriptional level, probably via regulation of −1857 to −1860 nt in Atg9B promoter and −1880 to −1870 nt in LAMP1 promoter, respectively.

## 4 DISCUSSION

It is of significance to explore new strategies, other than the current therapeutic methods, to improve the outcome of patients with advanced cervical cancer. The regulation of autophagy is being expected as potential therapeutics in clinical practice due to a key role of autophagy in the initiation and development of cancer and infectious disease. Cervical cancer is a malignant disease that results from high‐risk HPV persistent infection. To clarify the role of HPV in modulating autophagy, as well as involved mechanism, undoubtedly facilitates to explore targeting autophagy as a therapeutic approach to cervical cancer.

Previous studies have shown that the fate of oncogenic virus in host cells is largely dependent on cellular autophagic activity induced and maintained by infectious virus.^32^, ^33^, ^34^ However, the association of HPV with autophagy in cervical cancer is uncertain according to limited reports.^32^, ^33^, ^34^ We do not know whether HPV oncoprotein inhibits or induces autophagy, and whether autophagic activation promotes cellular homeostasis or lead to cell death in cervical cells, to date. In the study, we found that autophagosome synthesis is inhibited when 16E6/E7 was knocked down by specific siRNA in cervical cancer cells, and reversely, autophagosome formation and degradation was accelerated in HEK293 cells after 16E6 or 16E7 was forcibly overexpressed. Further, the proliferation of cervical cancer cells was inhibited and cell early apoptosis was promoted when autophagy inhibitor or blocker or RNAi was applied. Thus, our findings showed that 16E6/E7 possessed the ability to maintain proper‐activated autophagy via accelerating autophagic flux at the initiation and degradation stage in cervical cancer cells, consequently keeping the cell viability.

Transcriptome sequencing is an effective approach to find out molecular signaling pathway.^35^, ^36^, ^37^, ^38^, ^39^ To uncover the mechanism by which 16E6/E7 modulates autophagy in cervical cancer cells, we utilized transcriptome sequencing and bioinformatics analysis to search for downstream molecules that possibly participate in 16E6/E7 modulating autophagy in cervical cancer cells. As a result, Atg9B and LAMP1 were selected as candidate downstream molecules of 16E6/E7 among 586 differentially expressed genes between SiHa cells with and without 16E6/E7 knockdown. Atg9B, anchored in membranes, participates in autophagosomes formation and transportation,^40^, ^41^, ^42^, ^43^, ^44^ and LAMP1, a lysosomal‐associated protein, involves in autophagosomes degradation.^45^, ^46^ We found that LC3‐II was decreased after Atg9B knockout by double nickase plasmid and accumulated after LAMP1 knockdown by RNAi. We further confirmed by rescue experiment that autophagy inhibition caused by 16E6/E7 knockdown can be compensated, at least partially, by overexpressing Atg9B and/or LAMP1 in cervical cancer cells, suggesting that both Atg9B and LAMP1 were involved in 16E6/E7 modulating autophagy in cervical cancer cells. We further observed the interaction of viral oncogene with Atg9B and LAMP1, and found that 16E7 interacted with Atg9B, but not with LAMP1, by immunoprecipitation assay and Atg9B colocalized with LAMP1 by immunofluorescence assay. Our results suggest that the interaction between 16E7 and Atg9B facilitates autophagosomes formation and transportation to lysosome for degradation.

Unexpected, we did not find the interaction of 16E6 with Atg9B or LAMP1. However, 16E6/E7 knockdown inhibited the expression of Atg9B and LAMP1 in membranes, just as mentioned above, reminding us of a clue that 16E6, as a transcriptional factor,^30^, ^47^, ^48^, ^49^ modulates Atg9B and LAMP1 expression. Therefore, we utilized the JASPAR database, which is a database of transcriptional factor binding profile.^50^, ^51^, ^52^ As we expected, there were possible regulatory sites of 16E6 in Atg9B and LAMP1 promoter. Dual‐luciferase reporter system showed that −1750 to −2000 nt in Atg9B promoter and −1800 to −2000 nt in LAMP1 promoter were the most likely responsible sites of 16E6, suggesting that 16E6 modulates autophagy probably by controlling the expression of Atg9B and LAMP1 at the transcriptional level.

Taken our results together, HPV 16E6/E7 possesses the ability to maintain proper‐activated autophagy via accelerating autophagic flux at the initiation stage in cervical cancer cells, consequently keeping the cell viability. Both Atg9B and LAMP1 were involved in 16E6/E7 modulating autophagy, probably via E7 interacting with Atg9B directly and LAMP1 indirectly, and E6 upregulating Atg9B and LAMP1 expression, respectively. Our findings suggest that targeting autophagy may have a potential approach in cervical cancer therapeutics.
