# Supplementary figures and images for: Human papillomavirus 16E6/E7 activates autophagy via Atg9B and LAMP1 in cervical cancer cells
Source: Cancer Med. 2019 Jun 18;8(9):4404–16. doi: 10.1002/cam4.2351 (PMC6675746; doi:10.1002/cam4.2351)

Supplementary Figure 1

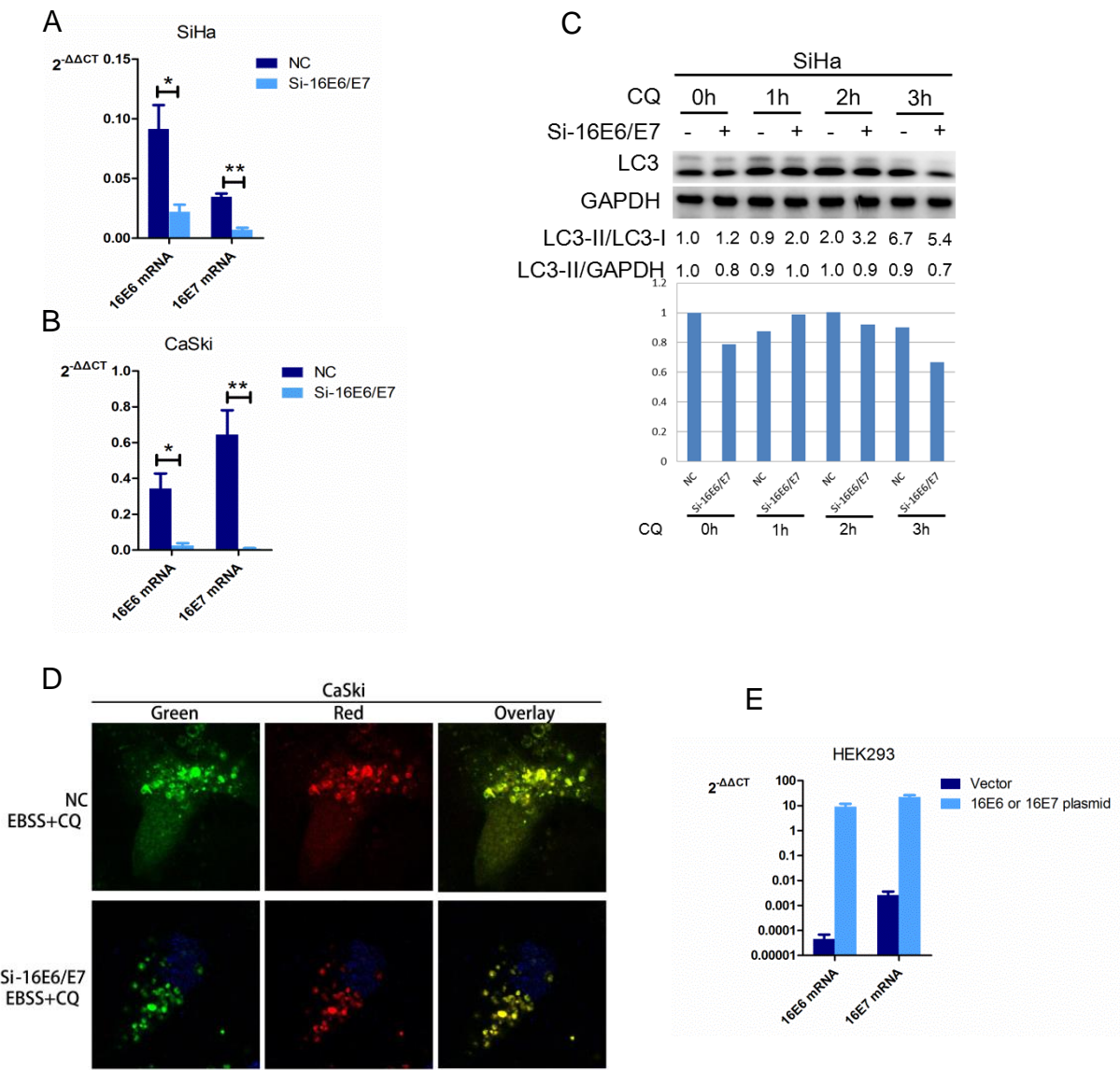

Supplement: Supplementary file 1 [file CAM4-8-4404-s001.pdf]

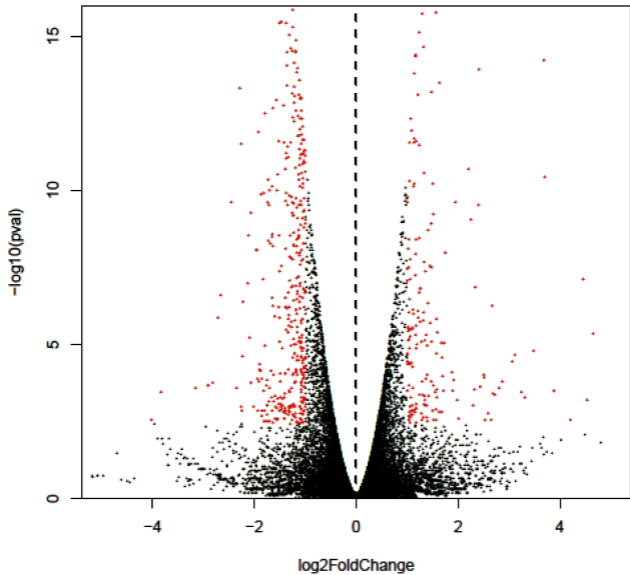

Supplement: Supplementary file 2 [file CAM4-8-4404-s002.pdf]

Statistics of Pathway Enrichment

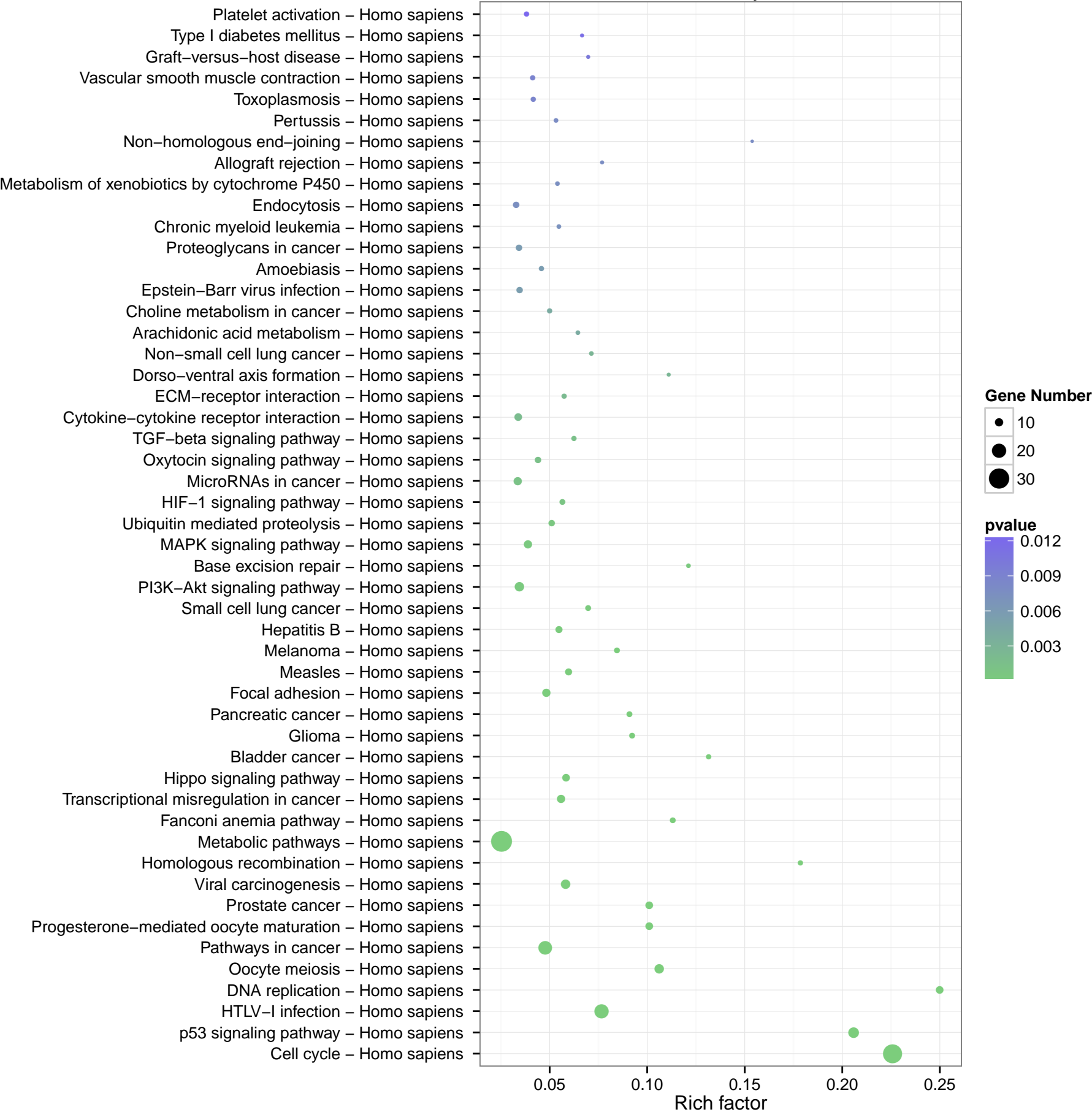

Supplement: Supplementary file 4 [file CAM4-8-4404-s004.pdf]

Gene Function Classification(GO)

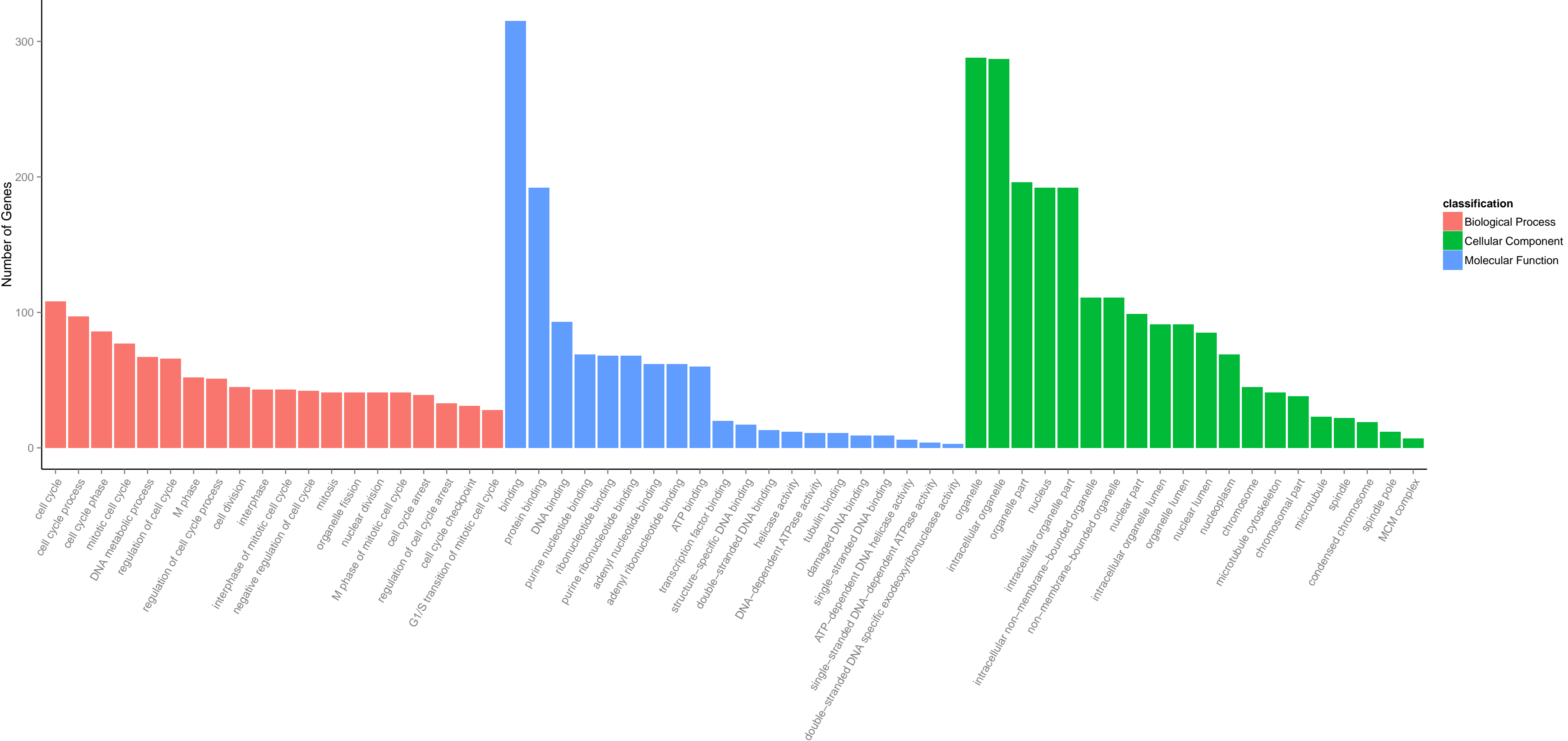

Supplement: Supplementary file 5 [file CAM4-8-4404-s005.pdf]

# Supplementary Figure 6

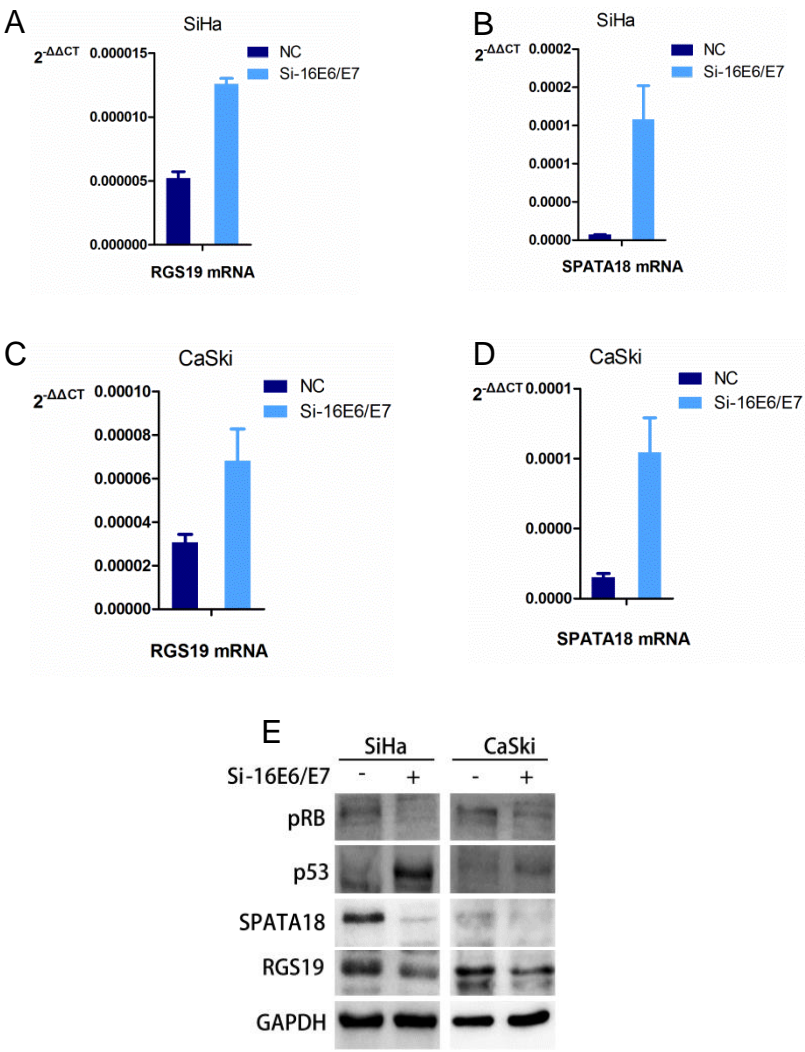

Supplement: Supplementary file 6 [file CAM4-8-4404-s006.pdf]

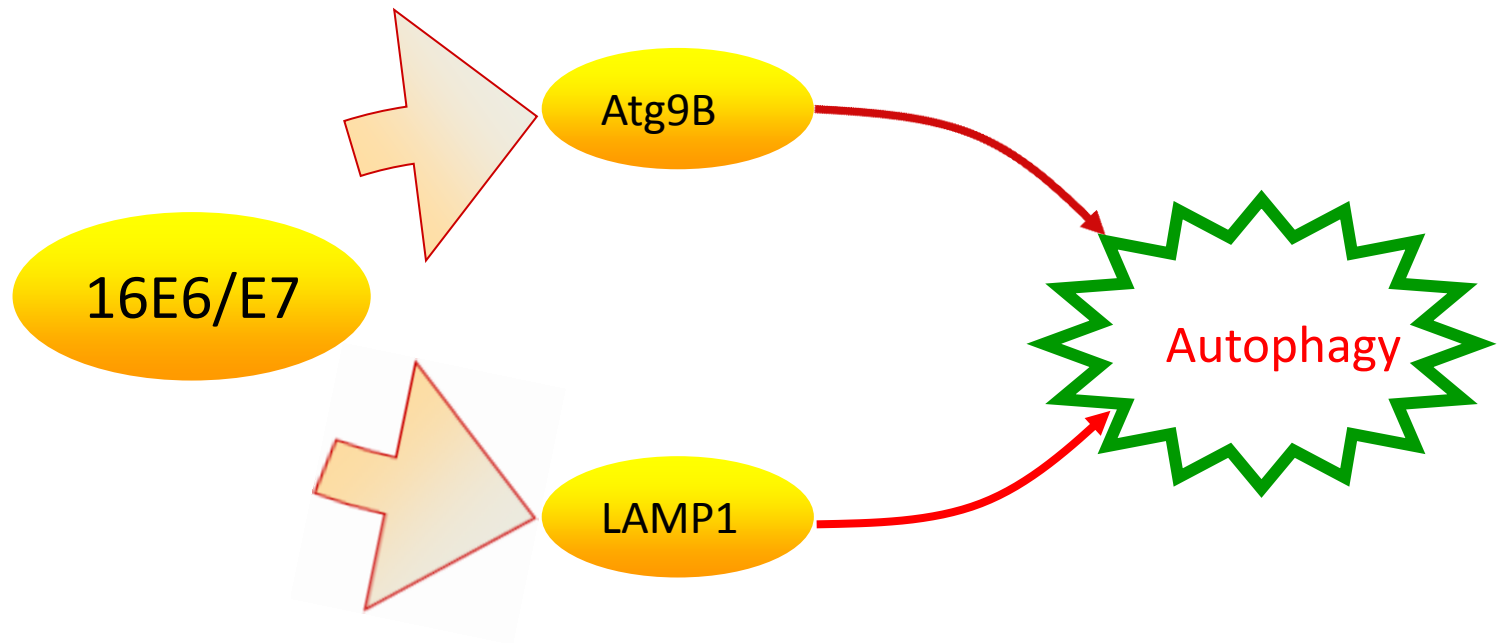

Supplement: Supplementary file 7 [file CAM4-8-4404-s007.pdf]
