# Supplementary material for: Human papillomavirus 16E6/E7 activates autophagy via Atg9B and LAMP1 in cervical cancer cells
Source: Cancer Med. 2019 Jun 18;8(9):4404–16. doi: 10.1002/cam4.2351 (PMC6675746; doi:10.1002/cam4.2351)
Supplement: Supplementary file 3 [file CAM4-8-4404-s003.pdf]

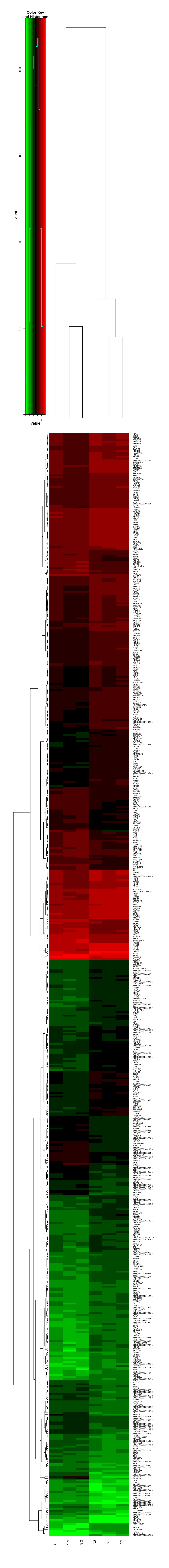
Color Key
and Histogram
Count
Value
Si1
Si3
Si2
N2
N1
N3
