# Supplementary material for: Human papillomavirus 16E6/E7 activates autophagy via Atg9B and LAMP1 in cervical cancer cells
Source: Cancer Med. 2019 Jun 18;8(9):4404–16. doi: 10.1002/cam4.2351 (PMC6675746; doi:10.1002/cam4.2351)
Supplement: Supplementary file 8 [file CAM4-8-4404-s008.pdf]

Table 1 Sequences of Si-RNA

Gene	Sequence (5'-3')
16E6/E7	GGUUGUGCGUACAAAGCAC
Atg5	GGAAUAUCCUGCAGAAGAATT
Atg16L1	CAGGACAATGTGGATACTCAT
LAMP1(1)	AGAAAUGCAACACGUUA
LAMP1(2)	GGAGAAGGAUAUUUACUGATT

Table 2 Sequences of primers

Gene	Sequence(5'-3')	
GAPDH	F-Primer	GACAGTCAGCCGCATCTTCT
	R-Primer	TTAAAAGCAGCCCTGGTGAC
16E6	F-Primer	CTGCAAGCAACAGTTACTGC
	R-Primer	GGCTTTTGACAGTTAATACACC
16E7	F-Primer	CATGGAGATACACCTACATTGC
	R-Primer	CACAACCGAAGCGTAGAGTC
SPATA18	F-Primer	GCAGGAAAAGCTAGACTTCTGG
	R-Primer	TGGCAACTTGCTCAATGAGTTC
RGS19	F-Primer	CTCGGAGGAGGAGGACTGTGATG
	R-Primer	GCTCCTACTCTGGAAGAAGT
Atg9B	F-Primer	TGTGCTCACCGTCTACGAC
	R-Primer	GGGAGGTAGTGCATGTGGG
LAMP1	1-F-Primer	TCTCAGTGAACTACGACACCA
	1-R-Primer	AGTGTATGTCCTCTTCCAAAAGC
	2-F-Primer	CAGATGTGTTAGTGGCACCCA
	2-R-Primer	TTGGAAAGGTACGCCTGGATG
	3-F-Primer	CACGAGAAATGCAACACGTTAC
	3-R-Primer	GGGTGCCACTAACACATCTGTAT

Table 3 Binding sites predicted in Atg9B promoter region by JASPAR database

	Score	Relative score	Start	End	Predicted Site Sequence
1	4.916	0.809768937	-18	-13	TGGAGA
2	5.559	0.851241457	-249	-244	CTTTGG
3	4.916	0.809768937	-284	-279	TGGAGA
4	6.636	0.899054901	-339	-334	TTTTGT
5	4.612	0.851919542	-383	-377	GCACACA
6	2.359	0.818757447	-383	-377	GCACACA
7	3.54	0.806106336	-383	-377	GCACACA
8	6.049	0.803726205	-385	-378	AAGCACAC
9	2.294	0.817567114	-387	-381	ACAAGCA
10	6.429	0.878792745	-387	-381	ACAAGCA
11	11.267	0.948903448	-541	-534	AAGTGTGA
12	12.104	0.948872414	-541	-534	AAGTGTGA
13	10.832	0.922402194	-541	-534	AAGTGTGA
14	10.878	0.947929172	-541	-534	AAGTGTGA
15	9.769	0.925653921	-541	-534	AAGTGTGA
16	6.112	0.830868167	-542	-537	CAAGTG
17	5.399	0.826577865	-546	-541	ATTACA
18	5.74	0.807786596	-649	-643	TTTGTTG
19	6.028	0.851117049	-678	-673	ATGAAA
20	6.158	0.85384061	-715	-710	TGTGTG
21	6.158	0.85384061	-717	-712	TGTGTG
22	6.158	0.85384061	-719	-714	TGTGTG
23	6.112	0.830868167	-843	-838	CAAGTG
24	6.636	0.899054901	-899	-894	TTTTGT
25	6.636	0.899054901	-917	-912	TTTTGT
26	6.232	0.855227295	-1029	-1023	AGCAGAA
27	4.612	0.851919542	-1065	-1059	GCACACA
28	2.359	0.818757447	-1065	-1059	GCACACA
29	3.54	0.806106336	-1065	-1059	GCACACA
30	4.636	0.812679271	-1102	-1097	CTCTAC
31	5.559	0.851241457	-1177	-1172	CTTTGG
32	4.846	0.806575056	-1644	-1639	TGGGCA
33	5.36	0.854724071	-1644	-1639	TGGGCA
34	8.415	0.902776786	-1694	-1687	TAATGGGC
35	6.636	0.899054901	-1842	-1837	TTTTGT

Table 4 Binding sites predicted in LAMP1 promoter region by JASPAR database

	Score	Relative score	Start	End	Predicted site sequence
1	4.208	0.800592934	-430	-425	CAGGAC
2	6.916	0.912472021	-610	-605	ACCCAC
3	2.377	0.819087078	-663	-657	ACAAACC
4	6.158	0.85384061	-696	-691	TGTGTG
5	7.097	0.919521008	-697	-692	CTGTGT
6	5.809	0.842573199	-784	-779	ATTAAC
7	5.307	0.822988668	-1234	-1229	ATAAAT
8	7.328	0.919913458	-1263	-1257	TTAATTA
9	5.809	0.842573199	-1387	-1382	ATTAAC
10	3.389	0.825982145	-1387	-1381	ATTAACT
11	4.704	0.803024561	-1519	-1513	ACAGCAA
12	6.322	0.858302063	-1685	-1679	AGAGAAA
13	5.803	0.842339121	-1689	-1684	ATCAAG
14	2.747	0.812366602	-1689	-1683	ATCAAGA
15	5.399	0.826577865	-1725	-1720	TTTAAA
16	5.331	0.843098552	-1877	-1872	AGCCAC
17	1.406	0.811163397	-1878	-1872	AAGCCAC
18	7.635	0.856711823	-1880	-1872	AAAAGCCAC
19	7.01	0.940512783	-1880	-1873	AAAAGCCA
20	4.846	0.806575056	-1909	-1904	TGGGAA
